# Genetic diversity analysis of cultivated and wild grapevine (*Vitis vinifera* L.) accessions around the Mediterranean basin and Central Asia

**DOI:** 10.1186/s12870-018-1351-0

**Published:** 2018-06-27

**Authors:** Summaira Riaz, Gabriella De Lorenzis, Dianne Velasco, Anne Koehmstedt, David Maghradze, Zviad Bobokashvili, Mirza Musayev, Goran Zdunic, Valerie Laucou, M. Andrew Walker, Osvaldo Failla, John E. Preece, Mallikarjuna Aradhya, Rosa Arroyo-Garcia

**Affiliations:** 1 Department of Viticulture and Enology, University of California, Davis, CA 95616 USA; 2 Department of Agricultural and Environmental Sciences, via Celoria 2, 20133 Milan, Italy; 3 Plant Sciences Department, UC Davis, Davis, CA 95616 USA; 4 USDA-ARS, National Clonal Germplasm Repository, University of California, Davis, CA 95616 USA; 5 Institute of Horticulture, Viticulture, and Oenology, Agricultural University of Georgia, Tbilisi, Georgia; 6 Department of Fruit Crops, Genetic Resources Institute, Azerbaijan National Academy of Sciences, AZ1106, Baku, Azerbaijan; 7 Institute for Adriatic Crops and Karst Reclimation, Split, Croatia; 8 INRA, UMR AGAP, F-34060 Montpellier, France; 9 Dpto. Biotecnología, CBGP-INIA, Campus de Montegancedo, Autovía M40 km 38, Pozuelo de Alarcón, 28223 Madrid, Spain

**Keywords:** Domestication, Genetic structure, Microsatellite, *V. vinifera* subsp. *sativa*, *V. vinifera* subsp. *sylvestris*

## Abstract

**Background:**

The mountainous region between the Caucasus and China is considered to be the center of domestication for grapevine. Despite the importance of Central Asia in the history of grape growing, information about the extent and distribution of grape genetic variation in this region is limited in comparison to wild and cultivated grapevines from around the Mediterranean basin. The principal goal of this work was to survey the genetic diversity and relationships among wild and cultivated grape germplasm from the Caucasus, Central Asia, and the Mediterranean basin collectively to understand gene flow, possible domestication events and adaptive introgression.

**Results:**

A total of 1378 wild and cultivated grapevines collected around the Mediterranean basin and from Central Asia were tested with a set of 20 nuclear SSR markers. Genetic data were analyzed (Cluster analysis, Principal Coordinate Analysis and STRUCTURE) to identify groups, and the results were validated by Nei’s genetic distance, pairwise F_ST_ analysis and assignment tests. All of these analyses identified three genetic groups: G1, wild accessions from Croatia, France, Italy and Spain; G2, wild accessions from Armenia, Azerbaijan and Georgia; and G3, cultivars from Spain, France, Italy, Georgia, Iran, Pakistan and Turkmenistan, which included a small group of wild accessions from Georgia and Croatia. Wild accessions from Georgia clustered with cultivated grape from the same area (*proles pontica*), but also with Western Europe (*proles occidentalis*), supporting Georgia as the ancient center of grapevine domestication. In addition, cluster analysis indicated that Western European wild grapes grouped with cultivated grapes from the same area, suggesting that the cultivated *proles occidentalis* contributed more to the early development of wine grapes than the wild vines from Eastern Europe.

**Conclusions:**

The analysis of genetic relationships among the tested genotypes provided evidence of genetic relationships between wild and cultivated accessions in the Mediterranean basin and Central Asia. The genetic structure indicated a considerable amount of gene flow, which limited the differentiation between the two subspecies. The results also indicated that grapes with mixed ancestry occur in the regions where wild grapevines were domesticated.

**Electronic supplementary material:**

The online version of this article (10.1186/s12870-018-1351-0) contains supplementary material, which is available to authorized users.

## Background

*Vitis vinifera* L., the commonly cultivated grapevine, is one of the most widely grown fruit plants in the world [1]. It has subspecies with West Asiatic and European origins, and ranges from Central Asia to the Mediterranean Basin [2]. Within the genus *Vitis*, *V. vinifera* is the primary species used in the global wine industry, which occupied 7.5 million hectares in 2012 and produced more than 67 million tons of grapes (http://www.oiv.int/). Within this species, two subspecies have been described, *V. vinifera* subsp. *sylvestris*, which includes the wild populations, and *V. vinifera* subsp. *sativa*, which includes the cultivated varieties that resulted from the domestication of the wild relatives [3]. The main phenotypic traits that distinguish the subspecies are: flower sex (dioecious for wild populations and hermaphroditic, or rarely female, for cultivated grapevines); and the seed morphology (spherical seeds with a small beak for *sylvestris* and pyriform seeds with a well-developed beak for the domesticated cultivars) [4, 5]. The two subspecies form a genetic and taxonomic continuum without breeding barriers resulting in spontaneous hybrids where they occur sympatrically or paripatrically [6–12].

Pioneering work of Negrul [13] divided the grapevine cultivars into three groups or *proles*: *occidentalis*, *pontica* and *orientalis* depending on geographic distribution and morphological and ecological differences. Grapevines found in the wide area extending from eastern Georgia, Armenia, Azerbaijan, and the former Soviet republics in Central Asia to the Near East have clear distinguishing features and were placed in the *proles orientalis*. Negrul recognized two sub-*proles* within this main group: *caspica*, composed of ancient vines used for vinification before the advent of Islam (from CE 500–1100), and the *antasiatica* including table and raisin grape cultivars of more recent origin. Varietal ecotypes found from Georgia to the Balkans were designated *P. pontica* sub-*proles georgica* and sub-*proles balkanica*, respectively.

Grape domestication occurred about 8000 years ago, during the Neolithic Age and was closely related to advances in winemaking in the Near East and area around Northern Mesopotamia [14–16]. The dissemination of grapevines from the primary domestication center into neighboring regions of Europe and Northern Africa followed three main pathways, first toward Mesopotamia, reaching the Southern Balkans and East Mediterranean Basin (end of the fifth millennium BCE), then toward Sicily to Western Europe and, finally, domesticated grapes were introduced to Central Europe during the first millennium BCE [16]. Meanwhile, during the fourth century BCE grapevine cultivation reached Central Asia, and near the second century BCE domesticated grapes were introduced into China and Japan [14, 15].

The cultivated grape *V. vinifera* subsp. *sativa* has played an important economic and cultural role throughout human history in different parts of the world. However, its ancestor the European wild grape *V. vinifera* subsp. *sylvestris*, is close to extinction. To capture and maintain the existing genetic diversity, researchers from East and West European countries under the framework of COST Action FA1003 (East-West collaboration for grapevine diversity exploration and mobilization of adaptive traits for breeding) initiated efforts to collect and preserve germplasm from a wide range of countries, including regions where autochthonous germplasm had not been investigated by genetic and ampelographic methods [17, 18].

The wild relatives of crop species have great importance to breeders as unique sources of genetic variation for breeding programs [19]. Wild grapevines are normally found in riparian ravines where they have access to water and can climb into the tree canopies. One impact of increased human population pressure is the destruction of natural habitats of wild flora and rapid erosion of genetic diversity. There is urgent need to characterize and conserve this valuable germplasm for future generations, and to design a strategy to preserve this species ex situ through extensive collections of wild grape that capture the genetic variation present in the Mediterranean basin and Central Asian regions. A closer analysis of Central Asian collections revealed that many genotypes resist fungal disease, such as downy mildew (*Plasmopara viticola*), powdery mildew (*Erysiphe necator*), and black rot (*Guignardia bidwellii*); all of which were supposedly introduced from North America about 150 years ago [20]. Other studies found that plants of *V. vinifera* subsp. *sylvestris* located in an area of Spain with heavy metal contamination exhibited high tolerance to copper stress [21]. Biotic and abiotic stresses from new pathogens, pests and a changing climate have spurred the creation of better-adapted varieties. Adequate genetic variation is the key to breeding crops capable of resisting these challenges.

Molecular analysis has provided insights into the genetic diversity of *V. vinifera* in relation to wild relatives, the genealogy of cultivars and the specific alleles linked to selected traits [15, 22, 23]. Although Central Asia is one of the centers of grapevine diversity, the majority of information about this region’s germplasm has emerged from accessions maintained in European and USA germplasm repositories [10, 12, 24]. The genotyping of wild and cultivated accessions from a broad range of viticultural areas at two large grapevine repositories provided a significant dataset capable of elucidating relationships within and between the two subspecies at the global level [10, 25]. Results from these studies suggest that grapevine spread from East-to-West after the first domestication process. The results also provide evidence of introgression from local *sylvestris* individuals with cultivated accessions [25], and the impact on genetic structure related to geographic origin and human use [10].

A limitation of previous examinations of grape genetic diversity was unbalanced sampling resulting in a germplasm collection set that was limited to one or more countries and was not broadly representative. In addition, the *sylvestris* and wild germplasm from the Caucasus Mountains and Central Asia was poorly represented or not analyzed in these studies. Although genetic, archeological and linguistic evidence suggests that southern Anatolia was the cradle of grape domestication, Transcaucasian remains a serious candidate as evidenced by ancient grape remains that were excavated from Neolithic archaeological sites in Azerbaijan as well as in Georgia [5]. Therefore, the results of previous studies may not present a complete picture of relationships between the wild and cultivated grapevine groups in that region and their association with the rest of world. The first large-scale characterization of both wild and domesticated grapevines, was done by Imazio et al. [12], utilizing SSR (Simple Sequence Repeats) fingerprint data from a set of 382 wild and 130 cultivated grapevine samples collected from Georgia. The results found four genetic groups, two for wild accessions and two for cultivated genotypes. The accessions from Georgia were included in a separate clade that highlighted the uniqueness of Georgian germplasm. Two other studies of grape germplasm from the Caucasus region also found that both wild and cultivated grapes had high genetic and morphological diversity [26, 27].

A previous study by Bacilieri et al. [10] analyzed genetic diversity of 2096 cultivated genotypes maintained in the Vassal germplasm collection and suggested the original center of grapevine domestication extended into many Central Asian countries. A comprehensive study that includes samples from the wild and cultivated groups, collected from opposing sides of an East-West gradient, and samples from Central Asian countries would provide a better understanding of the impact of geography and human selection on grapevine domestication and adaptive introgression. It would further allow us to determine the overall relationships of germplasm within the centers of domestication and with their wild progenitors. With these objectives, data were pooled from six previous studies {Laucou et al. [7], De Andrès et al. [8], Imazio et al. [12], Riaz et al. [24], Biagini et al. [28], Zdunić et al. [29]} and new data were generated for wild accessions collected from Croatia, Georgia, Armenia and Azerbaijan, to develop a well-balanced set that represented both subspecies and provided maximum representation of key geographical regions [Mediterranean basin and Central Asia (Spain, France, Italy, Croatia, Georgia, Armenia, Azerbaijan, Iran, Turkmenistan and Pakistan)]. SSR data were analyzed to infer the genetic structure of populations in wild and cultivated grapevines and to determine the role of Central Asian grapevine germplasm in the diversification of the cultivated gene pool. Results are discussed with emphasis on the conservation of wild germplasm tolerant to biotic and abiotic stress and its use in breeding programs.

## Methods

### Plant materials

A total of 1378 wild (*V. vinifera* spp. *sylvestris*) and cultivated (*V. vinifera* spp. *sativa*) samples from Transcaucasia (Armenia, Azerbaijan and Georgia), the Caspian Sea region (Turkmenistan and Pakistan), and Europe (Croatia, France, Italy and Spain) were included in the study. Table 1 and Additional file 1: Table S1 present a detailed list of the analyzed accessions based on their geographical origin and habitats. This list includes 975 samples of *sativa* and *sylvestris* germplasm from France, the Iberian Peninsula, Georgia, Turkmenistan, Pakistan, Italy, and Croatia that were genotyped in previous studies by Laucou et al. [7], De Andrès et al. [8], Imazio et al. [12], Riaz et al. [24], Biagini et al. [28] and Zdunić et al. [29]. In this work, 403 new accessions of *V. vinifera* spp. *sylvestris* from Armenia, Azerbaijan, Georgia and Croatia were genotyped. The wild germplasm from Armenia, Azerbaijan, and Georgia was collected as seeds from female vines gathered on two different collection trips. Seedling plants from a total of 17 seed lots are maintained in the USDA National Clonal Germplasm Repository in Davis, California, USA. The *sylvestris* samples from Croatia were collected from plants located in their natural habitats mostly along the Krka and Neretva rivers in 2013. Care was taken to select plants that were dioecious and notes were made for the flower phenotype and leaf morphology [29]. The Spanish accessions collected from natural habitats are maintained in the “El Encín” germplasm repository (Madrid, Spain). The French *sylvestris* accessions are maintained in the INRA “Domaine de Vassal” germplasm collection, and the Italian [28] and Georgian [12] samples are maintained in the germplasm repository of the University of Milan (Milano, Italy).

**Table 1:** List of cultivated and wild accessions of *Vitis vinifera* (1378) grouped into countries based on their geographic origin and analyzed by 20 SSR markers. Number of samples for each country is presented in brackets

*V. vinifera* subsp. *sativa* (396)			*V. vinifera* subsp. *sylvestris* (982)	
Europe		Asia	Europe	Asia
Spain (145) ^a^		Georgia (112) ^d^	France (46) ^c^	Armenia (49)
Italy (34) ^b^		Turkmenistan and Pakistan (73)^e^	Italy (289) ^b^	Azerbaijan (292)
France (32)^c^			Croatia (6) ^f^	Georgia (46) ^d^
			Croatia (32)	Georgia (30)
			Spain (192) ^a^	
Total	211	185	565	417

^a^[8]

^b^[28]

^c^[7]

^d^[12]

^e^[24]

^f^[29]

### DNA extraction and genotyping

Total genomic DNA was extracted from young leaves using DNeasy Plant Mini Kits (Qiagen, Valencia, CA, USA). Genotyping was carried out by amplifying 20 nuclear SSR loci: VMC1b11, VMC4f3.1, VVIb01, VVIh54, VVIn16, VVIn73, VVIp31, VVIp60, VVIq52, VVIv37, VVIv67, VVMD21, VVMD24, VVMD25, VVMD27, VVMD28, VVMD32, VVMD5, VVMD7, VVS2 [7]. The amplifications were performed as reported in [7]. The amplified loci were detected on an automated ABI 3500 Genetic Analyzer (Applied Biosystems, Life Technologies, Foster City, CA, USA). Allele sizes were scored using GeneMapper 4.0 software (Applied Biosystems, Life Technologies) and recorded in base pairs.

### Data analysis

#### Determination of flower phenotype

The flower phenotype of the *V. vinifera* subsp. *sylvestris* samples collected from Armenia, Azerbaijan, Georgia and Croatia was determined by a combination of a specifically designed marker from gene APT3 (adenine phosphoribosyl transferase) that is capable of distinguishing female plants from males or hermaphrodites [24]. We also used a specific allele of the SSR marker VVIb23 that is closely linked with the sex locus on chromosome 2, and is capable of distinguishing hermaphrodites from female or male plants. The VVIb23 locus polymorphism has been detected and reported in [30]. A total of 403 accessions were analyzed with these two markers to assign flower phenotype. The flower phenotypes of additional wild accessions from other countries were determined either during the time of collection or from plants maintainted in the germplasm repositories.

#### Genetic diversity

In order to combine the fingerprint data of new genotypes with previous data sets [7, 8, 12, 24, 28, 29], genetic profiles of eight reference cultivars (Cabernet Sauvignon, Chardonnay, Dolcetto, Pinot noir, Riesling, Thompson Seedless, Zinfandel, and Sangiovese) were used as references to standardize the allele calls.

The genetic diversity among groups and over all the groups was estimated. The normalized SSR genotyping data were used to determine the number of different alleles (Na), the effective number of alleles (Ne), Shannon’s Information Index (I), observed heterozygosity (Ho) and expected heterozygosity (He; [31]). The parameters were estimated by GenAlEx 6.5 software [32]. Weir and Cockerham’s F-statistics (F_IS_, F_IT_, F_ST_; [33]) per locus and F_IS_ values per each population were detected via FSTAT 2.9.3 and Arlequin 3.5.2.2 softwares, respectively [34, 35] *p*-values were evaluated over 1000 permutations. Allelic richness (AR) and private allelic richness (PAR) for each population were estimated using the rarefaction method, which compensates for differences in sample size (i.e. rarified allelic richness) among populations as implemented in HP-Rare 1.1 [36]. The effective number of migrants per generation (Nm) among the 12 grapevine populations and between the two subspecies was estimated using the private allele method of Barton and Slatkin [37] (1986) using GENEPOP 3.4 software [38].

#### Genetic relationships and differentiation

*Poppr* [39] package implemented in R 3.1 software [40] was used to design a phylogenetic tree with Neighbor-Joining. The distance matrix used in *Poppr* was calculated based on the Nei’s distance [41]. The unrooted dendrogram was plotted with the R package *ape* [42]. To measure how well the hierarchical structure from the dendrogram represents the actual distances, the cophenetic correlation coefficient (CCC) has been calculated performing the cophenetic function implemented in R software. Hclust R function was used to perfume hierarchical clustering using a neighbor-joining agglomeration method. In order to elucidate the genetic relationships within and among geographic groups, principal coordinate analysis (PCoA) was performed on the multilocus microsatellite data, which was then arranged into geographic groups using the package *adegenet* implemented in R [43]. Clustering validation and multivariate analysis was carried out using pairwise Nei’s genetic distance [44] and pairwise F_ST_ in GenAlEx 6.5 software. Finally, an analysis of molecular variance (AMOVA, [45]) was performed to characterize the partition of the observed genetic variation among and within populations and genetic groups using Arlequin 3.5.2.2 software. The significance test was performed over 1000 permutations.

#### Analysis of population structure

The microsatellite data were subjected to a Bayesian model-based cluster analysis using STRUCTURE 2.0 [46] to determine the optimum number of genetically supported groupings*.* STRUCTURE allocates individuals into a number of clusters (*K*) independent of population information based on genotypic data, so as to minimize deviations from Hardy-Weinberg and linkage equilibrium. The program uses a Markov Chain Monte Carlo (MCMC) procedure to estimate P(*X*|*K*), the posterior probability that the data fit the hypothesis of *K* clusters. The analysis assigns individuals to each of the *K* clusters based on the membership coefficient (*Q*-value), which sums to unity over the number of clusters (*K*) assumed. STRUCTURE was set to ignore population information, and to use an admixture model with correlated allele frequencies as it is considered to be the best option for subtle population structure [47]. The degree of admixture, alpha, was allowed to be inferred from the data. Alpha is close to zero when most individuals are from one population or another, while alpha is greater than one when most individuals are admixed [48]. The allele frequency parameter (lambda) was set to one. During a pilot study, it was found that a burn-in and MCMC (Markov Chain Monte Carlo) simulation lengths of 100,000 replicate runs were optimum to produce accurate parameter estimates. The number of clusters (*K*) varied from 2 to 10, and 20 replicate runs were carried out to quantify the variation of the likelihood for each *K*. The *K* value that provides the maximum likelihood (Ln *P*(*D*) in STRUCTURE) across runs is generally inferred as the most probable number of clusters. Nevertheless, the interpretation of *K* should be treated with care as it merely provides an ad hoc approximation [46] and sometimes genuine and fine population structure may be missed by STRUCTURE. Therefore, we used an ad hoc statistic *ΔK* to choose the optimum number of clusters (*K*) based on the second order rate of change in the log probability of data between successive *K* values as proposed by Evanno et al. [48].

## Results

### Flower phenotype in the wild accessions

Flower sex phenotype and seed morphology are key criteria normally used to differentiate subsp. *sylvestris* (dioecious vines, seeds with short beaks) from cultivated *sativa* forms (predominantly hermaphroditic flowers, seeds with larger beaks). The search for wild accessions was focused on collecting dioecious individuals because most cultivated genotypes are hermaphrodites. Flower phenotype data from the wild samples from Spain and Italy were recorded in the field and previously reported by Benito et al. [49] and Biagini et al. [28, 50]. The *sylvestris* samples from France, Georgia (University of Milan repository) and Croatia were collected from natural habitats and flower phenotypes were recorded based on the presence of fruit (female) and flower rachis without fruit (male) during collection. Only samples that met the basic dioecious phenotypic profile and leaf morphology of wild grapevines were included in the study. The flower phenotype of the subsp. *sylvestris* accessions collected from Armenia, Azerbaijan and Georgia (USDA repository) could not be determined because these plants were maintained in small containers. A combination of two DNA markers was used to differentiate the male, hermaphrodite and female flower phenotype for the set of 403 accessions from Armenia, Azerbaijan, Georgia and Croatia (Additional file 2: Table S2). Field phenotypic observations for the 38 accessions from Croatia matched the flower phenotype predicted by DNA analysis. Flower phenotypes assessed by DNA-based flower sex markers and field phenotyping of the wild forms of all the accessions of *V. vinifera* subsp. *sylvestris* are presented in Additional file 2: Table S2.

### Genetic diversity for *sativa* and *sylvestris* germplasm

Genetic data from 20 SSR loci and across 1378 grapevine samples, originating from Asia to Europe (Table 1) and representing both subspecies of *V. vinifera* (*sativa* and *sylvestris*), were used in this study. Additional file 1: Table S1 provides the allelic profiles of all analyzed samples. The number of alleles ranged from 11 for VVIq52 to 38 for VMC4f3.1 with an average of 20.95 alleles/locus. The number of effective alleles ranged from 2.192 for VVIn73 to 7.004 for VVIp31 with an overall average of 4.651. Both observed and expected heterozygosity varied greatly among loci and results of the fixation index with most loci suggested high levels of inbreeding (Table 2). The He values ranged from 0.477 (VVIn73 locus) to 0.803 (VVS2), with a mean value equal to 0.678. While, the Ho values varied from 0.535 (VVIn73) to 0.845 (VVIp31) and the mean overall value was 0.742. The locus with the lowest F value was VVIb01 (0.021), while the highest was VVIq52 (0.189). The mean F value for the dataset was 0.088.

**Table 2:** Diversity indices^*^ calculated for 1378 distinct genotypes including *sativa* and *sylvestris* accessions from Asia to Europe

Locus	Na^a^	Ne^b^	He^c^	Ho^d^	F^e^
VMC1b11	22	5.159	0.631	0.779	0.183
VMC4f3.1	38	5.970	0.776	0.810	0.041
VVIb01	20	3.261	0.662	0.681	0.021
VVIh54	25	4.213	0.665	0.747	0.116
VVIn16	14	2.551	0.538	0.566	0.054
VVIn73	14	2.192	0.477	0.535	0.120
VVIp31	25	7.004	0.790	0.845	0.065
VVIp60	20	4.581	0.703	0.758	0.071
VVIq52	11	2.862	0.519	0.634	0.189
VVIv37	21	5.694	0.667	0.792	0.153
VVIv67	26	5.314	0.719	0.790	0.089
VVMD21	18	2.617	0.490	0.571	0.138
VVMD24	12	3.754	0.666	0.720	0.072
VVMD25	23	4.987	0.760	0.789	0.035
VVMD27	20	4.576	0.678	0.767	0.117
VVMD28	31	5.960	0.724	0.819	0.115
VVMD32	19	5.017	0.734	0.785	0.061
VVMD5	20	5.142	0.766	0.800	0.042
VVMD7	20	5.595	0.785	0.804	0.023
VVS2	20	6.576	0.803	0.839	0.045
Mean	20.950	4.651	0.678	0.742	0.088

^*a^No. of allele per locus

^b^No. of effective alleles

^c^Expected Heterozygosity

^d^Observed Heterozygosity

^e^Fixation Index

Allelic profiles were used to calculate statistical indices and determine the genetic diversity of the cultivated and wild genotypes (Table 3). The number of alleles per locus (Na) was 9.120 for *sativa* and 9.164 for *sylvestris* samples. The Italian cultivars had the lowest Na value (4.900) of the cultivated accessions and the highest Na value (12.600) was detected in the Georgian cultivars. The number of alleles per locus for the wild accessions varied between 7.050 (Armenia) and 12.850 (Georgia). The Ne value over the whole dataset was 4.441. The *sativa* accessions from Italy (3.688) and *sylvestris* accessions from France (2.792) had the lowest Ne values. The highest Ne values were detected in cultivated accessions (5.751) and wild individuals (6.016, Table 3) from Georgia. Within *sativa*, the allelic richness, adjusted to a minimum sample size of 42 genes, ranged from 6.200 alleles for Spanish accessions to 9.330 for Italian accessions, with an overall mean of 7.848 alleles across loci. Within the *sylvestris* accessions, allelic richness ranged from 5.870 for the Armenian group to 10.200 for the Georgian group with an overall mean of 7.089 across loci. The private allelic richness for *sativa* ranged from 0.020 for the Spanish and French groups to 0.520 for the Italian and Turkmenistan/Pakistani groups with an overall mean frequency of 0.314 alleles across loci. Within *sylvestris,* this richness ranged from 0.020 for the Azerbaijani accessions to 0.980 for Georgian wild grapes with an overall mean of 0.344 private alleles per locus. The mean Shannon’s Information Index (I) value for the wild accessions was slightly lower than that for the cultivars (1.60 vs. 1.641), with an overall value of 1.619 (Table 3). In general, the Ho values were lower than He values for each group, except for cultivated samples from France (0.765 vs. 0.708) and Italy (0.798 vs. 0.682). The Ho value for *sativa* was higher than *sylvestris* (0.754 vs. 0.649), while the overall mean value (0.692) was more similar to the *sylvestris* value than the *sativa* value. The He value for *sativa* (0.735) was higher than the *sylvestris* value (0.722).

**Table 3:** Genetic diversity estimates in wild and cultivated grapevines for each analyzed population. Results are arranged based on the geographical origin and habitat

Populations	N^a^	Na^b^	Ne^c^	AR^d^	PAR^e^	I^f^	Ho^g^	He^h^	F_IS_^i^
France	25.750	6.900	4.035	6.720	0.020	1.516	0.765	0.708	0.057 ***
Georgia	103.100	12.600	5.751	8.530	0.490	1.877	0.746	0.776	0.066 ***
Italy	6.600	4.900	3.688	9.330	0.520	1.349	0.798	0.682	-0.166
Spain	144.500	10.350	4.650	6.200	0.020	1.670	0.730	0.739	0.022 ***
Turkmenistan, Pakistan	71.000	10.850	5.290	8.460	0.520	1.793	0.723	0.768	0.053 ***
Overall *sativa*	70.190	9.120	4.682	7.848	0.314	1.641	0.754	0.735	0.039 ***
Armenia	47.150	7.050	3.967	5.870	0.100	1.506	0.676	0.718	-0.077
Azerbaijan	278.450	8.550	3.649	5.980	0.020	1.476	0.650	0.694	0.095 ***
Croatia	36.850	9.650	4.849	8.260	0.880	1.779	0.658	0.759	-0.038 ***
France	45.650	6.350	2.792	5.912	0.143	1.202	0.591	0.604	0.035 **
Georgia	73.800	12.850	6.016	10.200	0.980	1.999	0.653	0.815	0.138 ***
Italy	289.000	10.250	4.044	6.410	0.160	1.569	0.660	0.709	0.055 ***
Spain	192.000	9.450	4.556	6.990	0.130	1.686	0.655	0.755	0.131 ***
Overall *sylvestris*	137.557	9.164	4.268	7.089	0.345	1.602	0.649	0.722	0.169 ***
Overall Loci and Pops	109.488	9.146	4.441	7.405	0.332	1.619	0.692	0.727	0.151 ***

^a^No. of samples; ^b^No. of alleles per locus; ^c^No. of effective alleles; ^d^Allelic Richness; ^e^Private allele richness; ^f^Shannon's Information Index; ^g^Observed heterozygosity; ^h^Expected heterozygosity, ^i^Inbreeding coefficient within individuals relative to the subpopulation; **p ≤ 0.10; ***p ≤ 0.05 calculated over 1000 permutations

The samples were arranged in 12 groups based on their origin and subspecies, and F_IS_ values were calculated (Table 3). The values ranged from − 0.166 (Italian *sativa* samples) to 0.138 (Georgian *sylvestris* samples). The values for the *sylvestris* populations were generally higher than the *sativa* populations. Among the wild accessions, populations from Georgia and Spain had the highest F_IS_ values (0.138 and 0.131, respectively). The populations of cultivated accessions with the highest inbreeding coefficient were from France (0.057) and Georgia (0.066). The F_IS_ value over all loci and populations was 0.151 and the *sativa* value was lower than that for *sylvestris* (0.039 versus 0.169). Most of the F_IS_ values had a *p*-value lower than 0.1.

### Cluster analysis

**Fig. 1:**
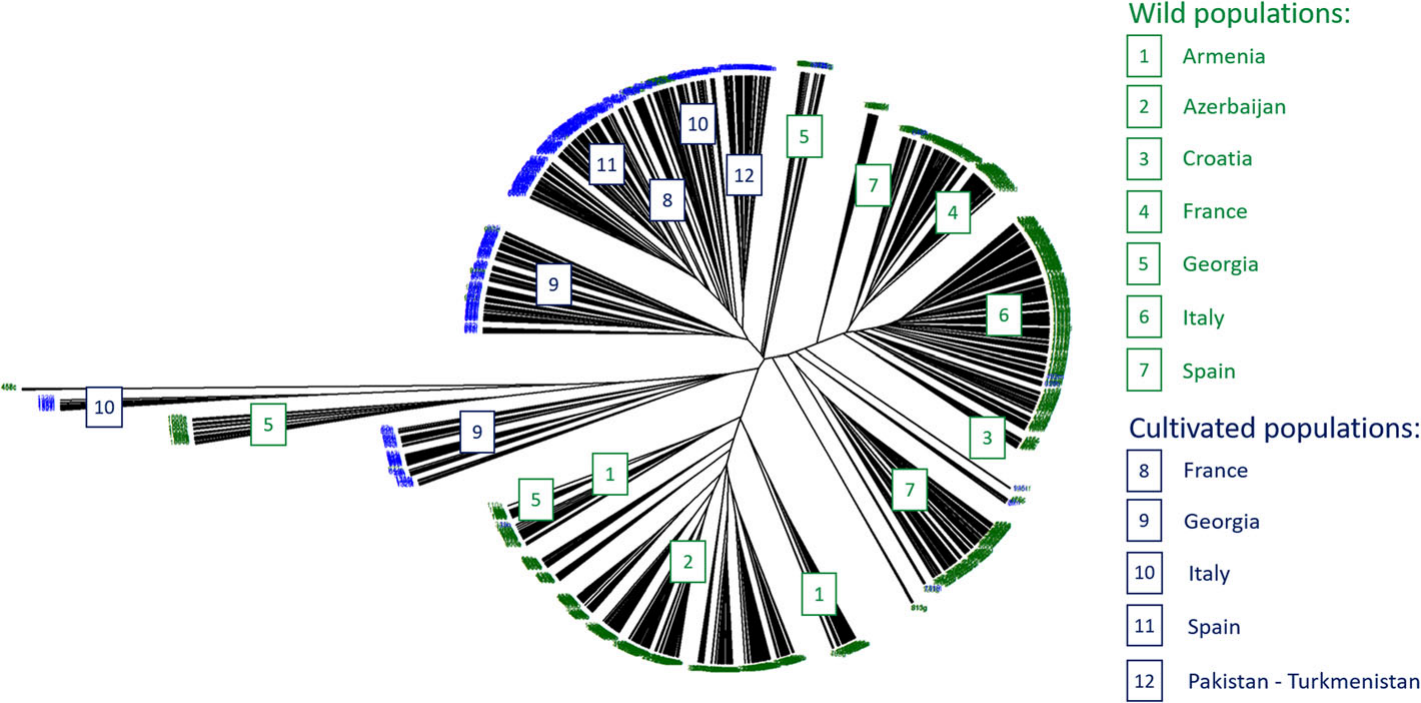
NJ dendrogram showing relationships among 1378 cultivated and wild grapevine accessions obtained by data analysis from 20 SSR loci. Samples are arranged based on their origin and membership in the sativa and sylvestris subspecies

The neighbor-joining (NJ) cluster analysis based on the pair-wise distance matrix showed clear differentiation between the two subspecies (Fig. 1). A number of wild individuals clustered with the cultivated samples and vice versa. The dendrogram showed three main groups with cophenetic correlation coefficient (CCC) value of 0.75 (Fig. 1). The *sylvestris* accessions divided into two groups and *sativa* accessions formed a third major group. The first group of wild germplasm contained most of the Transcaucasian *sylvestris* accessions from Armenia (#1), Azerbaijan (#2) and Georgia (#5) and the second group consisted of the European wild accessions from Croatia (#3), France (#4), Italy (#6) and Spain (#7). The Spanish wild accessions were further split into two groups, one of them including the French wild samples (#4). There were two sub-groupings within the *sativa* cluster, one containing the French (#8), Italian (#10), Spanish (#11) and Turkmenistan-Pakistan samples (#12), and the other containing some of the Georgian samples (#9). Two additional minor clusters were identified, both containing Georgian samples. One of these contained the wild samples (#5) and the other both wild and cultivated samples (#5 and #9). The latter cluster also contained a small group of Italian cultivars (#10).

### Population structure analysis and differentiation

**Fig. 2:**
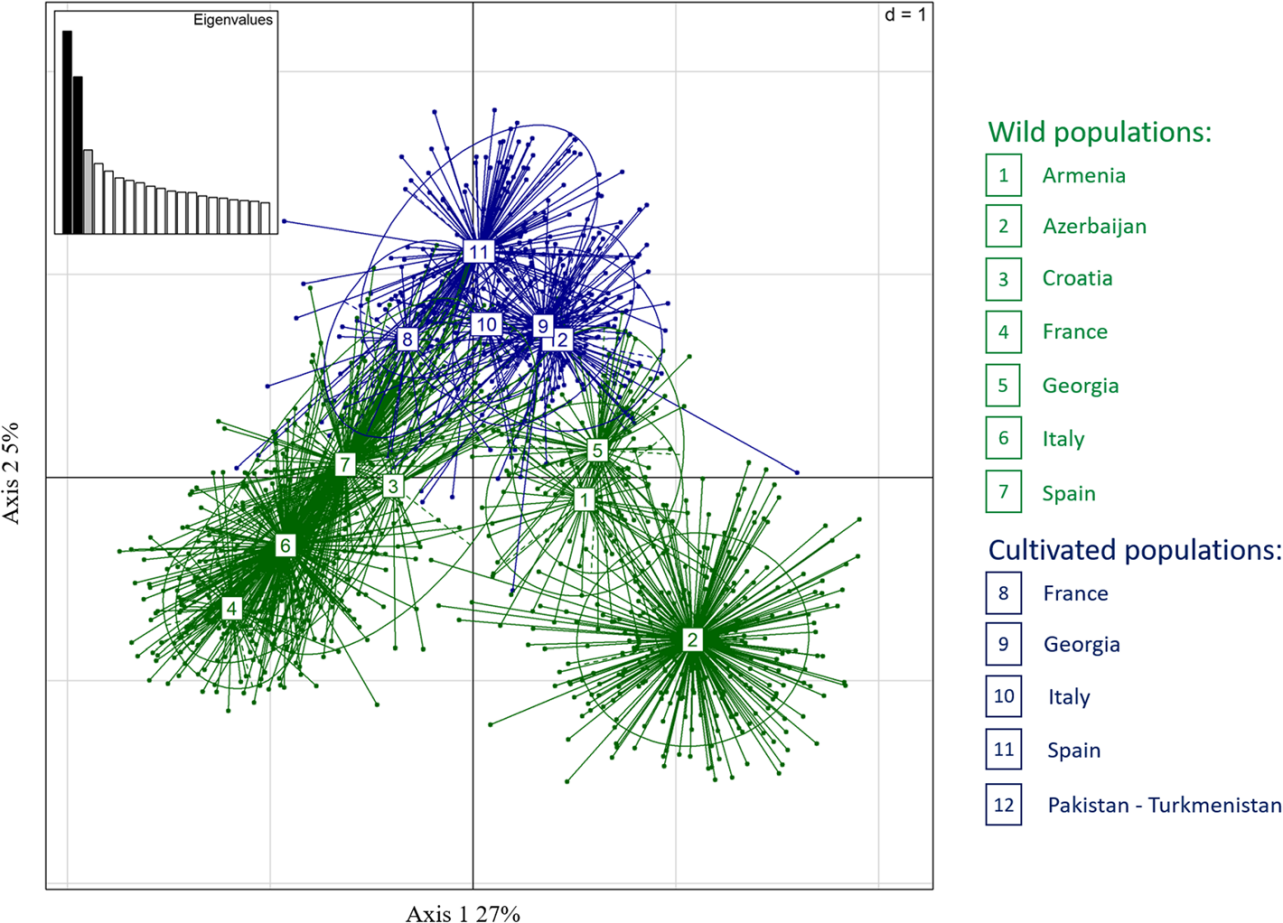
Relationships between wild and cultivated grapevine genotypes (1378) as represented by the first two principal coordinates of a PCoA using allelic profiles from 20 SSR molecular markers. Samples are arranged based on their origin and membership in the sativa and sylvestris subspecies

**Fig. 3:**
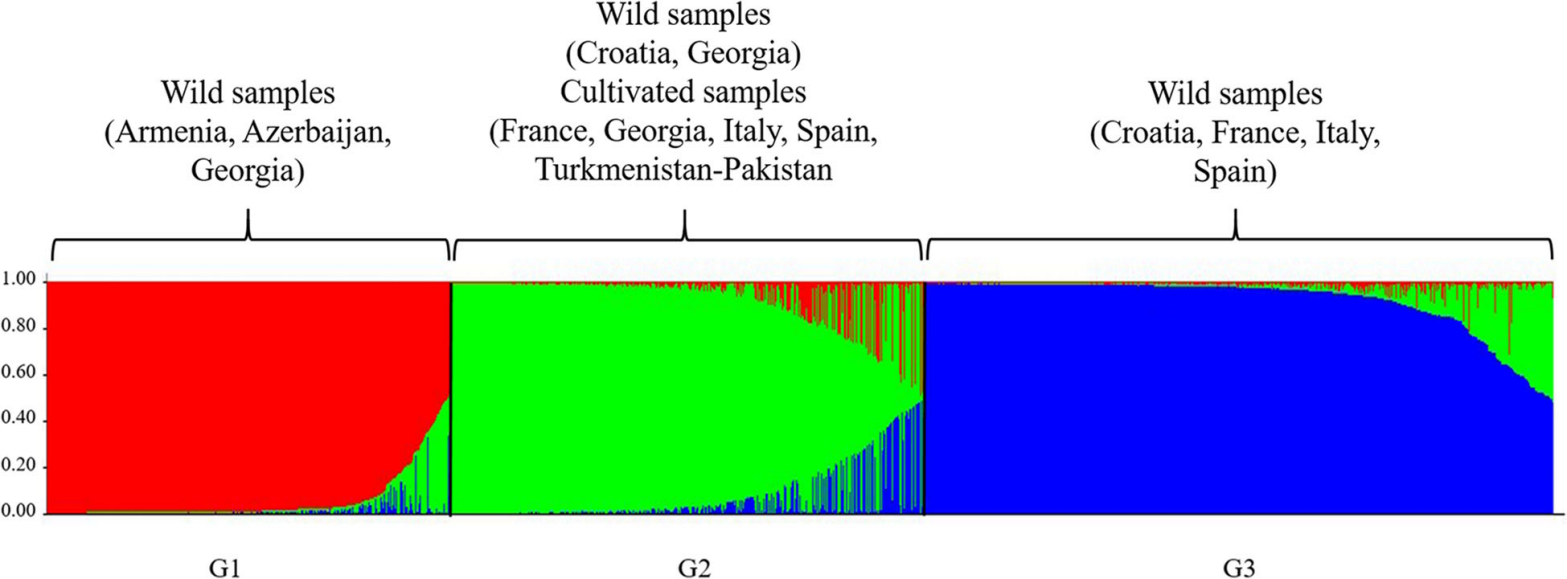
Barplot displaying the admixture proportions of wild and cultivated grapevine genotypes as estimated by STRUCTURE analysis at K = 3 and 7. The Evanno’s ΔKs statistics indicated K = 3 as the best supported level of population subdivision using simulation model with K values ranging from 2 to 10

In order to identify the structure of populations and the correlations among samples, two different analyses were performed. PCoA was based on the genetic distance matrix obtained by the SSR profiles. Projections of the PCoA were plotted in a 2-dimension scatter plot (Fig. 2). The PCoA 2D projection of the first two principal axes accounted for ~ 32% of the total molecular variation (Fig. 2). Significant differentiation between the two subspecies and the European and Transcaucasian *sylvestris* groups was observed. The *sylvestris* samples from Armenia (#1), Azerbaijan (#2) and Georgia (#5) were clearly differentiated from the rest of the *sativa* and *sylvestris* groups. The European *sylvestris* groups (#3, #4, #6 and #7) formed overlapping clusters, as did the accessions from Armenia (#1) and Georgia (#5). All five groups of *sativa* from Europe (#8, #10 and #11), Georgia (#9), Turkmenistan and Pakistan (#12) were closely associated. The *sativa* groups were closely associated with *sylvestris* accessions from Europe (#3, 4, 6, 7) and Transcaucasia (#1, 5), with the exception of the *sylvestris* accessions from Azerbaijan (#2). There was large variability within each of these groups and subspecies. The second method used to evaluate the relationship among genotypes was a clustering algorithm implemented in the program STRUCTURE. The Bayesian analysis results of genetic structure for the wild (*sylvestris*) and cultivated grapevines (*sativa*) were roughly comparable with the NJ cluster analysis and PCoA results, but STRUCTURE did not detect subtle differentiation among some of the populations. The estimated log probability values [Ln Pr (X|K)] for different K gradually increased reaching a maximum value at K = 3 with non-significant variation among replicate runs, beyond which the rate of increase between successive K decreased and variance among runs increased (Fig. 3). Plotting the second order rate of change of the log probability of data (∆K) with respect to the number of clusters, against K predicts the true K according to Evanno et al. [48], and such analysis produced a clear peak at K = 2, but the second order rate of change of likelihood distribution showed that the rate of change is bigger between K = 3 and 4, therefore, K = 3 is the most likely number of clusters in the genetic structure of these grape populations. About 84% of genotypes were assigned to a cluster at K = 3, with a percentage of assignment higher than 80%. The proportion of admixed genotypes was about 16% (Additional file 3: Table S3). Plotting the Q matrix values (the estimated membership coefficients for each individual in each K clusters) for K = 3 (Fig. 3), revealed clusters roughly corresponding to the two major groups within *sylvestris*, one from the Caucasus (Armenia, Azerbaijan and Georgia; G2) and the other from Europe (Croatia, France, Italy and Spain; G3), and one group with the French, Georgian, Italian and Spanish *sativa* accessions (G1). As observed in the NJ cluster analysis and PCoA, there were genotypes with mixed ancestry in all three groups. The populations with the highest percentage of admixed samples were Armenia (39%) and Georgia (49%) for wild groups and France (32%) for sativa accessions (Additional file 3: Table S3).

Population structure among the 12 tested populations, irrespective of the subspecies, was summarized by the Wright’s F-statistics (F_IT_, F_ST_ and F_IS_) (Additional file 4: Table S4). The VVMD21 locus had the highest value for F_IT_, F_ST_ and F_IS_ (0.380, 0.235 and 0.189, respectively), while the lowest F_IT_ and F_IS_ values were detected for the VMC4f3.1 locus (0.095 and 0.005, respectively), and VVMD25 had the lowest F_ST_ value (0.056). The number of migrants (Nm) after correction for sample size was 1.33, when samples were arranged in 12 populations. When the samples were arranged in two subpopulations (*sativa* and *sylvestris*), Nm was 4.88.

Nei’s genetic distance and F_ST_ were calculated to validate the results obtained from cluster analysis and PCoA. The pairwise values for the 12 geographic groups are listed in Table 4. Nei’s genetic distance had a wide range of values, from 0.116 recorded for the pairwise French and Spanish *sativa* samples, to 0.830 for the *sylvestris* samples from Georgia and France. The F_ST_ values varied from a low of 0.021 detected for the French and Spanish cultivated accessions to a high of 0.125 for the *sylvestris* individuals from Azerbaijan and France. Nei’s genetic distance and F_ST_ values for *sativa* and *sylvestris* groups were 0.159 and 0.023, respectively.

**Table 4:** Estimates of pairwise Nei’s genetic distance (below the diagonal) and F_ST_ values (above the diagonal) within overall wild and cultivated grapevine groups

	Armenia	Azerbaijan	Croatia	France (*sylvestris*)	France (*sativa*)	Georgia (*sylvestris*)	Georgia (*sativa*)	Italy (*sylvestris*)	Italy (*sativa*)	Spain (*sylvestris*)	Spain (*sativa*)	Turkmenistan, Pakistan
Armenia	–	**0.043**	**0.173**	**0.208**	**0.172**	**0.097**	**0.106**	**0.139**	**0.160**	**0.123**	**0.144**	**0.131**
Azerbaijan	0.268	**–**	**0.120**	**0.177**	**0.146**	**0.062**	**0.064**	**0.117**	**0.115**	**0.088**	**0.104**	**0.091**
Croatia	0.457	0.463	–	**0.189**	**0.164**	**0.086**	**0.069**	**0.116**	**0.076**	**0.003**	**0.061**	**0.067**
France (*sylvestris*)	0.721	0.730	0.363	–	**0.101**	**0.198**	**0.175**	**0.101**	**0.184**	**0.122**	**0.154**	**0.196**
France (*sativa*)	0.439	0.603	0.290	0.473	–	**0.175**	**0.141**	**0.084**	**0.150**	**0.080**	**0.128**	**0.180**
Georgia (*sylvestris*)	0.254	0.243	0.458	0.830	0.423	–	**0.054**	**0.108**	**0.096**	**0.085**	**0.079**	**0.061**
Georgia (*sativa*)	0.465	0.515	0.421	0.830	0.295	0.269	–	**0.100**	**0.050**	**0.049**	**0.058**	**0.050**
Italy (*sylvestris*)	0.409	0.533	0.213	0.262	0.291	0.469	0.471	–	**0.117**	**0.078**	**0.094**	**0.116**
Italy (*sativa*)	0.575	0.702	0.432	0.748	0.312	0.478	0.288	0.470	–	**0.066**	**0.066**	**0.066**
Spain (*sylvestris*)	0.576	0.565	0.289	0.286	0.303	0.544	0.502	0.247	0.501	–	**0.044**	**0.071**
Spain (*sativa*)	0.419	0.629	0.384	0.686	0.116	0.396	0.261	0.427	0.253	0.359	–	**0.056**
Turkmenistan, Pakistan	0.322	0.484	0.448	0.774	0.327	0.353	0.278	0.467	0.338	0.510	0.253	–

In bold, significant values with *p* ≤ 0.05, calculated over 1000 permutations

The AMOVA analysis is presented in Additional file 5: Table S5. When the total genetic variation was partitioned, 9.54% was attributed to the differences among populations, 6.68% to the differences among individuals within populations and 83.78% to the differences within individuals, with levels of significance estimated over 1000 permutations lower than 0.05. F_ST_, F_IS_ and F_IT_ parameters overall the loci and populations were 0.095, 0.073 and 0.162, respectively (*p* ≤ 0.05).

## Discussion

The main objective of this study was to analyze the pattern of genetic diversity within and between wild and cultivated grapes from the Mediterranean basin and Central Asia – considered to be the center of domestication. We pooled information from six previous studies that examined both wild and cultivated accessions, and genotyped an additiopnal 403 wild accessions from the Caucases region and Croatia at 20 microsatellite loci. The microsatellite marker data from 1378 accessions was subjected to NJ clustering and Bayesian methods to elucidate groupings of wild grapevine populations and to infer gene flow and gene frequency changes that occurred during domestication.

### Assessment of flower sex within *sylvestris* populations

Taxonomic distinctions between the two subspecies, *sylvestris* and *sativa*, are based on leaf morphology and the dioecious state of wild forms. According to the model of Antcliff [51], the flower phenotype is controlled by a single major locus with three alleles: male (M) dominant to hermaphrodite (H), which is dominant to the female (F). In the wild, only male and female vines exist in the absence of gene flow from hermaphroditic cultivated varieties. However, the possibility of hybridization and seed dispersion increases where wild vines are in close proximity to cultivated types. The wild accessions from earlier studies were collected with careful consideration of flower phenotype and leaf morphology [7, 8, 12, 28, 29]. The samples from Armenia, Azerbaijan, and Georgia were collected as seed lots. Analyses of flower phenotype based on linked markers found that the Georgia populations had more female than male vines, and that seed lot DVIT3357 consisted of only female and hermaphrodite vines indicating gene flow from cultivated to wild types (Additional file 2: Table S2). However, the Armenian, and Azerbaijan populations had a higher proportion of male plants. Heterogeneous plant sex distribution was also observed in earlier study of Spanish *sylvestris* samples [49] with a majority of the plants being male.

### Pattern of genetic diversity distribution within and among the subspecies

The two subspecies of *V. vinifera* included in this study exhibited high levels of polymorphism and heterozygosity across the 20 microsatellite loci and significant diversity was observed within and between the subspecies (Tables 2 and 3). This trend was expected in a divergent gene pool composed of subspecies and hermaphroditic cultivars that have undergone intensive human selection during domestication*.* Data obtained in other studies [10, 11] are similar to the results from our survey. Genetic diversity within and among the different geographic groups in both subspecies, as demonstrated by the effective number of alleles and allelic richness, suggests that there is significant diversity both within and between the subspecies (Table 3). The *sativa* and *sylvestris* accessions from Georgia had the highest number of effective alleles and allele richness suggesting that this region is the center of diversity for *V. vinifera* [2].

In general, we expected to see higher levels of heterozygosity in *sylvestris* because of its obligate out-crossing nature compared to its domesticated counterpart *sativa*. The Ho value of the *sativa* group appeared slightly higher than the He values; while the trend was the opposite for the *sylvestris* accessions. These differences correspond with the positive F_IS_ values in *sylvestris*, particularly in the populations from Spain and Georgia, which suggests a high level of genetic relationship among the individuals from the same wild populations (Table 3). Such matings can affect individual and population dynamics and increase inbreeding. However, the F_IS_ values of some wild populations were close to zero as expected in randomly mating populations (Table 3). These opposing results may be explained by random genetic drift of alleles among subpopulations due to sample size. The reduced level of diversity that we observed in *sylvestris* samples has also been noted in other studies [10–12]. The *sylvestris* accessions in many parts of the world are considered endangered and fragmented due to deforestation and urbanization. Man-made and natural geographical barriers can also lead to the isolation of wild populations in their native habitat, and could lead to significant inbreeding, reduced gene flow within and among different geographic groups and, hence, lower levels of heterozygosity.

The F_IS_ values were close to zero in the cultivated accessions suggesting random mating, except the Italian accessions. The negative F_IS_ values for Italian populations indicated an excess of heterozygotes, but it was not statistically significant (Table 3). The deficiency of homozygotes in the majority of the cultivated groupings suggests that they are made up of germplasm with divergent demographic (founder effects, bottlenecks, dispersal) and selection histories. Germplasm collections are usually mixtures of genotypes. Thus, geographic groups in these collections exhibit relatively high levels of differentiation, resulting in higher than expected levels of heterozygosity. This is commonly observed in woody perennial crops where cultivars are selected for their vigor, which indirectly favors high levels of heterozygosity [52–54].

The results of the AMOVA and F-stat analysis confirmed that high levels of diversity were present within populations, while low levels of genetic diversity were found among populations. These results are consistent with the findings from other studies [10–12].

### Genetic structure and differentiation within and between the subspecies

A significant differentiation within and between the two subspecies was detected by cluster analysis and PCoA (Figs. 1 and 2). Both analyses found clear differentiation between the Western European wild grapevines and the wild samples collected from the Caucasus. The French and Spanish wild grapes were closely allied and had a close genetic relationship. These results were in agreement with Arroyo et al. [22], who used chloroplast markers to find that these populations had the same haplotype. The Spanish wild grapevines showed hierarchical differentiation, suggesting that gene flow among neighboring populations caused a stepping-stone model of population structure. Alternatively, the hierarchical differentiation could be the result of climatic differences across diverse geographic regions. The Croatian *sylvestris* accessions were related to the European *sylvestris* individuals and formed a basal sister group indicating a common gene pool. The wild grapevines from Transcaucasia, including Armenia, Azerbaijan, and Georgia, formed a distinct sub-group that contained several accessions of Azerbaijani wild grapevines. Similarly, the Georgian and Armenian wild grapes split into two subgroups each, however they shared a common Transcaucasia gene pool. The *sylvestris* vines in the Transcaucasia region grow in a wide range of isolated habitats created by the Greater and Lesser Caucasus Mountain systems where they are differentially adapted to local environments [12, 54]. Some of the *sylvestris* individuals, both in Caucasian and European germplasm, clustered with the cultivated samples. These accessions are most likely feral hybrids of *sativa* and *sylvestris*, which may have been used in breeding programs or as cultivated selections (Figs 1 and 2).

Within *sativa*, two distinct groups of cultivars from Georgia were observed, one appeared as a sister clade of Italian, French and Spanish cultivars (Fig. 1), while the other group was closely related to an Italian *sativa* and Georgian *sylvestris* sub-group. This result could suggest that the first domesticated cultivars in Central Asia and Caucasus (*proles pontica*), left a genetic footprint in the Western European *proles occidentalis* accessions. This genetic kinship could also be a reflection of early breeding programs in the Mediterranean region where *sylvestris* or hybrid feral vines with superior fruit were utilized in crosses with domesticated lines.

The overall pattern of differentiation depicted by the PCoA is very similar to the NJ cluster analysis (Figs. 1 and 2). Clusters within *sylvestris* accessions from Georgia and Armenia overlapped and were closely associated with cultivated forms from Georgia, Pakistan and Turkmenistan. The close association of Georgian wild grapevines with Georgian cultivated accessions strongly supports their involvement in the initial domestication of grapevine [55–57]. Evaluation with NJ cluster analysis and PCoA, indicates that local European *sylvestris* vines might have contributed to the selection and introgression of genes into Western European grapevines in the later part of the domestication process (Fig. 2). The Bayesian STRUCTURE analysis supported differentiation among the major groups only, while the fine-scale differentiation between some of the groups, especially those with mixed ancestry, was not evident (Fig. 3). Bayesian inference of genetic structure indicated considerable gene flow with moderate differentiation between the two subspecies. These results suggest that wine grape cultivation and wine making promoted the domestication of wild grapevines, creation of new varieties, and advancement of growing techniques early in grapevine’s history. Further introgression and mixing of wild germplasm in localized communities would have contributed to the high proportion of grapevines with mixed ancestry. Interestingly, analyses of ancestry values of tested western cultivars identify some with a high ancestry values in Group 3 (Additional file 3: Table S3). These grapevine cultivars correspond to the Spanish cultivars; Albariño, Caiño Blanco, Ferrón, Maturana, Ondarrabi Betlza and the European cultivars Arvine Petite, Cot, Chenin Blanc, Petit Verdot. Pinot Meunier and Sauvignon Blanc. These cultivars have been described as more closely related to wild accessions [8] and our results support the introgression of western *sylvestris* into some of the current Western European cultivars.

It is difficult to suggest that wild grape forms homogeneous populations considering the vast geographic expanse and the often fragmented and isolated populations that occur under heterogeneous climatic conditions. However, our results suggest Georgia as an ancient center of grapevine domestication with its wild grapes closely related to the cultivated grapes of the same region (*proles pontica*), and Western European (*proles occidentalis*). This observation confirms earlier studies that suggested that *proles pontica* were gradually introduced by human migration towards Western Europe [10, 25, 58, 59]. Cluster analysis shows a relationship between Western European wild grapes and cultivated grapes, suggesting that *proles occidentalis* grapevines contributed to the early development of wine grapes to a much greater extent than the wild vines from Eastern Europe. Previous studies using SNPs markers [25] proposed a Near East origin of *vinifera* and presented evidence of introgression from local *sylvestris* as the grape moved into Europe, but the degree to which local Western European wild *sylvestris* genetically contributed to Western European *vinifera* cultivars remains a contentious issue. Our results suggest and support at least two separate domestication events that gave raise to cultivated grape; one derived from the Transcaucasia wild grape, and another from the wild grapes of Western Europe.

Scientific interest in the highly endangered ancestor of cultivated grapevine, *V. vinifera* subsp*. sylvestris*, has centered on questions of conservation genetics, and deepening our understanding of the domestication history of the cultivated crop [22]. However, since domestication traits such as higher yield, larger berries, higher sugar content are often accompanied by a loss of resistance to abiotic and biotic stress, it is beneficial to search for such factors in the wild forms of the crop’s ancestors. In fact, salt-tolerant grape accessions can be found in the North African *sylvestris* population [60], and the recent identification of wild and cultivated accessions from Germany, Iran and Georgia with tolerance to mildew diseases supports the potential of this wild ancestor as a genetic resource for disease resistance breeding [24, 61–63]. Given that wild Eurasian and North Africa wild *V. vinifera* germplasm and Asian *Vitis* germplasm are largely unexplored, their identification, preservation, and characterization for biotic and abiotic resistance and berry quality [64, 65] traits are very important for the future of the wine and grape industry.

## Conclusions

The two sub-species of *V. vinifera*, subsp. *sativa* and subsp. *sylvestris*, are distinct based on analysis of SSR data, but extensive gene flow was observed in regions where these two taxa came in contact. Our results suggest that Georgia is an ancient center of grape domestication based on a genetic affinity between wild accessions from Georgia and cultivated grapes from Georgia (*pontica*) and Western Europe (*occidentalis*). Results also suggest that Western European wild grapes were related to cultivated grapes, and that Western European *sylvestris* contributed to the development of Western European wine grapes. The results also support at least two separate domestication events that gave raise to cultivated grape; one derived from the Transcaucasian wild grape and another from the wild grape of Western Europe.

Finally, wild grape germplasm can contribute many useful traits such as resistance to damaging pests and diseases, and better adaptation to climate change. Thus, we must intensify efforts to collect, characterize and preserve not only the Western and Eurasian wild *V. vinifera* germplasm, but also *sylvestris* accessions from North Africa and Central Asia. These wild grape relatives will have a key role in future grape and wine industry.

## Additional files

Additional file 1: **Table S1.** SSR allelic profile of 1378 sativa and sylvestris grapevine samples genotyped at 20 SSR molecular markers. (XLSX 643 kb)

Additional file 2: **Table S2.** Determination of flower phenotype and genotype. The APT3 gene marker distinguished females (F) from males (M) or hermaphrodites (H). SSR marker VVIb23 is tightly linked to the flower sex locus and the unique allele 282 is linked to hermaphrodism. (XLSX 57 kb)

Additional file 3: **Table S3.** Ancestry values (mean and standard deviation values over 20 iterations) for the genetic groups inferred by STRUCTURE analysis of 1378 sativa and sylvestris grapevine samples genotyped at 20 SSR loci. (XLSX 145 kb)

Additional file 4: **Table S4.** Locus-wise genetic differentiation parameter comparisons of 12 populations from both *V. vinifera* subspecies sativa and sylvestris. (DOCX 19 kb)

Additional file 5: **Table S5.** Results of the AMOVA analysis carried out among and within 12 populations of wild and cultivated grapevine. (DOCX 16 kb)

## Abbreviations

AMOVA: Analysis molecular of variance
BCE: Before current era
CE: Current era
F: Female
F: Inbreeding coefficient
Fst: Fixation index
H: Hermaphrodite
He: Expected heterozygosity
Ho: Observed heterozygosity
M: Male
N: Sample size
Ne: Number of effective alleles
PCoA: Principal coordinate analysis
SNP: Single Nucleotide Polymorphism
SSR: Simple sequence repeat
UMIL: University of Milan
USDA: United States Department of Agriculture
