# Supplementary material for: Genetic diversity analysis of cultivated and wild grapevine (Vitis vinifera L.) accessions around the Mediterranean basin and Central Asia
Source: BMC Plant Biol. 2018 Jun 27;18:137. doi: 10.1186/s12870-018-1351-0 (PMC6020434; doi:10.1186/s12870-018-1351-0)
Supplement: Supplementary file 4 — Table S4. Locus-wise genetic differentiation parameter comparisons of 12 populations from both V. vinifera subspecies sativa and sylvestris. (DOCX 19 kb) [file 12870_2018_1351_MOESM4_ESM.docx]

**Table S4.** Locus-wise genetic differentiation parameter comparisons of 12 populations from both *V. vinifera* subspecies *sativa* and *sylvestris*.

| **Locus** | **F_IT_^a^** | **F_ST_^b^** | **F_IS_^c^** | **Sig_a^d^** | **Sig_b^e^** | **Sig_w^f^** |
| --- | --- | --- | --- | --- | --- | --- |
| VMC1b11 | 0.211 | 0.086 | 0.137 | 0.074 | 0.107 | 0.676 |
| VMC4f3.1 | 0.095 | 0.091 | 0.005 | 0.078 | 0.004 | 0.774 |
| VVIb01 | 0.124 | 0.094 | 0.033 | 0.069 | 0.021 | 0.638 |
| VVIh54 | 0.202 | 0.148 | 0.064 | 0.126 | 0.047 | 0.68 |
| VVIn16 | 0.153 | 0.110 | 0.049 | 0.075 | 0.029 | 0.578 |
| VVIn73 | 0.215 | 0.162 | 0.063 | 0.094 | 0.031 | 0.453 |
| VVIp31 | 0.146 | 0.105 | 0.046 | 0.096 | 0.038 | 0.78 |
| VVIp60 | 0.185 | 0.169 | 0.019 | 0.149 | 0.014 | 0.715 |
| VVIq52 | 0.275 | 0.153 | 0.144 | 0.107 | 0.085 | 0.506 |
| VVIv37 | 0.230 | 0.140 | 0.105 | 0.121 | 0.078 | 0.666 |
| VVIv67 | 0.147 | 0.089 | 0.064 | 0.078 | 0.052 | 0.753 |
| VVMD21 | 0.380 | 0.235 | 0.189 | 0.171 | 0.105 | 0.451 |
| VVMD24 | 0.164 | 0.107 | 0.064 | 0.084 | 0.045 | 0.662 |
| VVMD25 | 0.097 | 0.056 | 0.044 | 0.046 | 0.034 | 0.738 |
| VVMD27 | 0.196 | 0.094 | 0.112 | 0.075 | 0.081 | 0.643 |
| VVMD28 | 0.195 | 0.099 | 0.107 | 0.088 | 0.085 | 0.716 |
| VVMD32 | 0.220 | 0.208 | 0.016 | 0.193 | 0.012 | 0.726 |
| VVMD5 | 0.158 | 0.127 | 0.036 | 0.112 | 0.028 | 0.741 |
| VVMD7 | 0.120 | 0.105 | 0.017 | 0.090 | 0.013 | 0.757 |
| VVS02 | 0.144 | 0.098 | 0.051 | 0.089 | 0.041 | 0.777 |

^a^ The inbreeding coefficient within individuals relative to the total; ^b^ The inbreeding coefficient within subpopulations relative to the total; ^c^ The inbreeding coefficient within individuals relative to the subpopulation; ^d^ component of variance among samples; ^e^ component of variance among individuals within samples, ^f^ component of variance within individuals respectively.
