# Supplementary material for: Genetic diversity analysis of cultivated and wild grapevine (Vitis vinifera L.) accessions around the Mediterranean basin and Central Asia
Source: BMC Plant Biol. 2018 Jun 27;18:137. doi: 10.1186/s12870-018-1351-0 (PMC6020434; doi:10.1186/s12870-018-1351-0)
Supplement: Supplementary file 5 — Table S5. Results of the AMOVA analysis carried out among and within 12 populations of wild and cultivated grapevine. (DOCX 16 kb) [file 12870_2018_1351_MOESM5_ESM.docx]

**Table S5**. Results of the AMOVA analysis carried out among and within 12 populations of wild and cultivated grapevine.

| **Source of variation** | **Degree of**  **Freedom** | **Sum of**  **Squares** | **Variance components** | **Percentage of variation (%)** | **Fst^a^** | **Fis^b^** | **Fit^c^** |
| --- | --- | --- | --- | --- | --- | --- | --- |
| Among Populations | 11 | 1793.8 | 0.719 *** | 9.54 |  |  |  |
| Among Individuals within  populations | 1366 | 10004.5 | 0.503 *** | 6.68 |  |  |  |
| Within Individuals | 1378 | 8704.5 | 6.316 *** | 83.78 |  |  |  |
| **Total** | 2755 | 20502.9 | 7.539 | 100 | 0.095 *** | 0.073 *** | 0.162 *** |

^a^ The inbreeding coefficient within individuals relative to the subpopulation; ^b^ The inbreeding coefficient within individuals relative to the total; ^c^ The inbreeding coefficient within subpopulations relative to the total. *** *p* ≤ 0.05 estimated over 1000 permutations.
